# Evolutionary refugia and ecological refuges: key concepts for conserving Australian arid zone freshwater biodiversity under climate change

**DOI:** 10.1111/gcb.12203

**Published:** 2013-04-18

**Authors:** Jenny Davis, Alexandra Pavlova, Ross Thompson, Paul Sunnucks

**Affiliations:** Australian Centre for Biodiversity, School of Biological Sciences, Monash UniversityClayton, VIC, 3800, Australia

**Keywords:** arid environments, climate change, climatic decoupling, freshwater biodiversity, refuges, refugia

## Abstract

Refugia have been suggested as priority sites for conservation under climate change because of their ability to facilitate survival of biota under adverse conditions. Here, we review the likely role of refugial habitats in conserving freshwater biota in arid Australian aquatic systems where the major long-term climatic influence has been aridification. We introduce a conceptual model that characterizes evolutionary refugia and ecological refuges based on our review of the attributes of aquatic habitats and freshwater taxa (fishes and aquatic invertebrates) in arid Australia. We also identify methods of recognizing likely future refugia and approaches to assessing the vulnerability of arid-adapted freshwater biota to a warming and drying climate. Evolutionary refugia in arid areas are characterized as permanent, groundwater-dependent habitats (subterranean aquifers and springs) supporting vicariant relicts and short-range endemics. Ecological refuges can vary across space and time, depending on the dispersal abilities of aquatic taxa and the geographical proximity and hydrological connectivity of aquatic habitats. The most important are the perennial waterbodies (both groundwater and surface water fed) that support obligate aquatic organisms. These species will persist where suitable habitats are available and dispersal pathways are maintained. For very mobile species (invertebrates with an aerial dispersal phase) evolutionary refugia may also act as ecological refuges. Evolutionary refugia are likely future refugia because their water source (groundwater) is decoupled from local precipitation. However, their biota is extremely vulnerable to changes in local conditions because population extinction risks cannot be abated by the dispersal of individuals from other sites. Conservation planning must incorporate a high level of protection for aquifers that support refugial sites. Ecological refuges are vulnerable to changes in regional climate because they have little thermal or hydrological buffering. Accordingly, conservation planning must focus on maintaining meta-population processes, especially through dynamic connectivity between aquatic habitats at a landscape scale.

## Introduction

Freshwater ecosystems are experiencing declines in biodiversity far greater than those in terrestrial systems because of multiple and interacting factors. These include overextraction, water pollution, flow modification, destruction and degradation of habitats and invasion by exotic species (Dudgeon *et al*., 2006). The fact that global climate change represents an additional, all-encompassing threat to freshwater biodiversity, and aquatic ecosystem structure and function has been clearly articulated (IPCC, 2007a; Vörösmarty *et al*., 2010; Woodward *et al*., 2010). All freshwater ecosystems are considered vulnerable to climate change because their relative isolation and physical fragmentation within terrestrial landscapes mean that many species will have limited ability to disperse as temperatures increase (Woodward *et al*., 2010). The biota of arid zone freshwater habitats are especially vulnerable because of their isolation by large tracts of inhospitable intervening habitat and very little and infrequent hydrological connectivity. In addition, the potentially large indirect effects of increasing human demands for water are likely to be most profound in arid environments (Palmer *et al*., 2008) adding to the challenge of sustaining freshwater habitats. Here, we review the applicability of the concepts of refugia and refuges to freshwater systems in arid Australia to inform climate adaptation strategies for freshwater biodiversity conservation.

An integrated understanding of the historical processes (climatic, geological, hydrological, evolutionary and ecological) that have created the planet's major biomes is critical to managing them under future environmental change (Moritz, 1994; Byrne *et al*., 2008). Insights into the historical ecology of biota in arid biomes were recently provided by Byrne *et al*. (2008). Arid biomes or drylands (hyper-arid to subhumid regions, where rainfall is very low and potential evaporation very high) cover almost half of the world's land area (Brendock & Williams, 2000). Extensive arid regions occur in Australia and Africa and extend from the Middle East through Central Asia. Although aquatic ecosystems are not a major feature of arid regions they are prominent in some areas, including the high veldt of South Africa, parts of inland Australia, north-western Texas and contiguous parts of New Mexico, USA (Brendock & Williams, 2000). Our review is of relevance to all systems where past climatic change has been dominated by aridification.

## Approaches to identifying refugia

Keppel *et al*. (2012) proposed a habitat-based definition of refugia, as sites to which biota retreat, persist in and potentially expand from under changing environmental conditions. Dobrowski's (2011) focus on identifying refugia as places where local climate is decoupled from regional climate complements this approach. Groves *et al*. (2012) suggested protecting climatic refugia as part of a suite of approaches to climate change adaptation that could be integrated into existing or new biodiversity conservation plans. Distinguishing between macrorefugia and microrefugia is important, with the former defined as regions with favorable climates while the latter are small areas of favorable climate within a region of largely unfavorable climate (Provan & Bennett, 2008a; Rull, 2009; Ashcroft *et al*., 2012). Distinguishing whether refugia from future climate change are likely to be located within (*in situ*) or outside (*ex situ*) a species' current distribution, and whether refugia are based on climatic or habitat stability, are also important considerations (Ashcroft, 2010; Groves *et al*., 2012).

The desert pupfishes of southwestern USA are an important example of a species for which the presence of climatic refugia during the dry phases of the Pleistocene played a major role in the evolution and ecology of a desert biota. Many desert fishes are relict species and endemism is high (Rolston, 1991). US desert species occupy similar geomorphic habitats, have similarly restricted distributions and experience similar climatic influences to fishes in the Australian arid zone. The zoogeographic models of gene flow proposed for US desert fishes occupying refugia and other aquatic habitats by Meffe & Vrijenhoek (1988) appear to be of relevance to Australian species. Refugia have also been important in the evolution and ecology of aquatic organisms in extreme polar climates. Arctic lakes have acted as refugia for freshwater invertebrates, the cladoceran crustaceans *Daphnia* and *Leptodora* (Weider & Hobaek, 2003; Millette *et al*., 2011), and freshwater fishes, the lake trout, *Salvelinus* spp (Wilson & Hebert, 1996), during multiple Pleistocene glaciation events.

Unfortunately, much confusion exists over the use of the terms refugium and refuge despite a considerable increase in their use (Keppel & Wardell-Johnson, 2012). The term refuge is often used to indicate microhabitats that provide protection from contemporary temporal or spatial disturbances. In the ecological literature, ‘refugia’ have been defined as habitats that convey spatial and temporal resistance and/or resilience to biotic communities affected by disturbances (Sedell *et al*., 1990) or as places or times where the negative effects of disturbance are lower than those in the surrounding area (or time). Organisms have a higher chance of survival in ‘refugia’, these serve as a source for recruitment or recolonization to more severely affected areas when the disturbance abates (Lancaster & Belyea, 1997). The concept of a refuge is a relative one, being influenced by the spatial and temporal scale, the disturbance regime and species' adaptations (Magoulick & Kobza, 2003). For aquatic systems both the terms ‘refugium’ and ‘refuge’ have been widely used in relation to droughts, floods, high flows, thermal stress and predation (Sheldon *et al*., 2010).

Keppel *et al*. (2012) suggested that shorter ecological timescales of minutes to decades are the defining characteristic of ‘refuges’ while ‘refugia’ are based on longer evolutionary timescales of millennia. Using freshwater biota in arid landscapes as a model we sought to identify the evolutionary refugia that have enabled biota to persist over millennia of climatic change (aridification), as well as the ecological refuges providing shelter under the contemporary highly variable climatic regime. Distinguishing between refugia and refuges enables us to explore the processes that support the persistence of both, and their potential to act as refugial sites under future climatic change. To do this, we integrated pattern- and process-based information as advocated by Keppel *et al*. (2012) and Dobrowski (2011). We considered past climatic, geological and hydrological conditions combined with an understanding of biogeography and phylogeography of extant aquatic species. The climatic fluctuations over the Quaternary (2.6 mya-present), particularly the advance and retreat of the ice sheets through multiple glacial cycles, especially the last glacial maximum (LGM), had a major impact on the distribution of Northern Hemisphere species (Provan & Bennett, 2008b). In contrast, most of Australia did not support extensive Pleistocene ice sheets. However, significant climatic oscillations, characterized by warm/wet interglacial periods and cool/dry glacial maxima, did occur (Byrne, 2008).

Our focus is on fishes and aquatic invertebrates, the groups that are highly dependent on the presence of freshwater to complete all or most of their life cycles. We did not include frogs due to the ability of desert-dwelling species to aestivate underground in extremely dry conditions for many years. Waterbirds were not included because flight provides them with the mobility to move large distances between extant arid and mesic zones depending upon water availability.

## The Australian arid zone biome

**Fig. 1:**
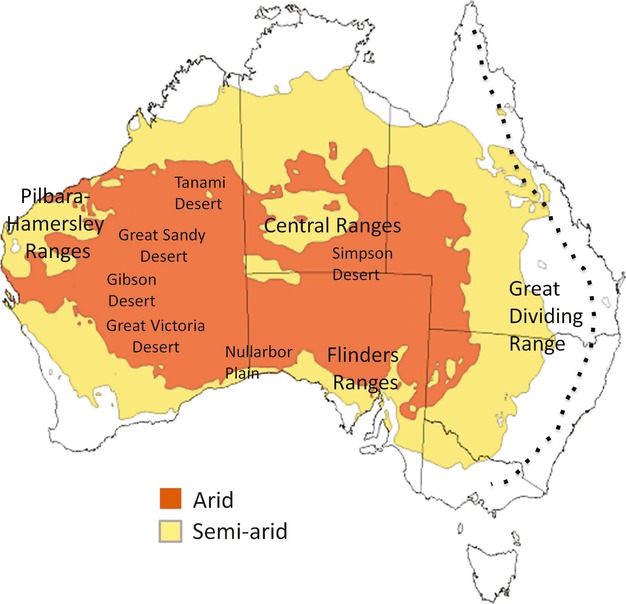
Extent of the arid and semiarid regions comprising the Australian arid biome and the location of major ranges and deserts. Source: http://www.bom.gov.au.

**Fig. 2:**
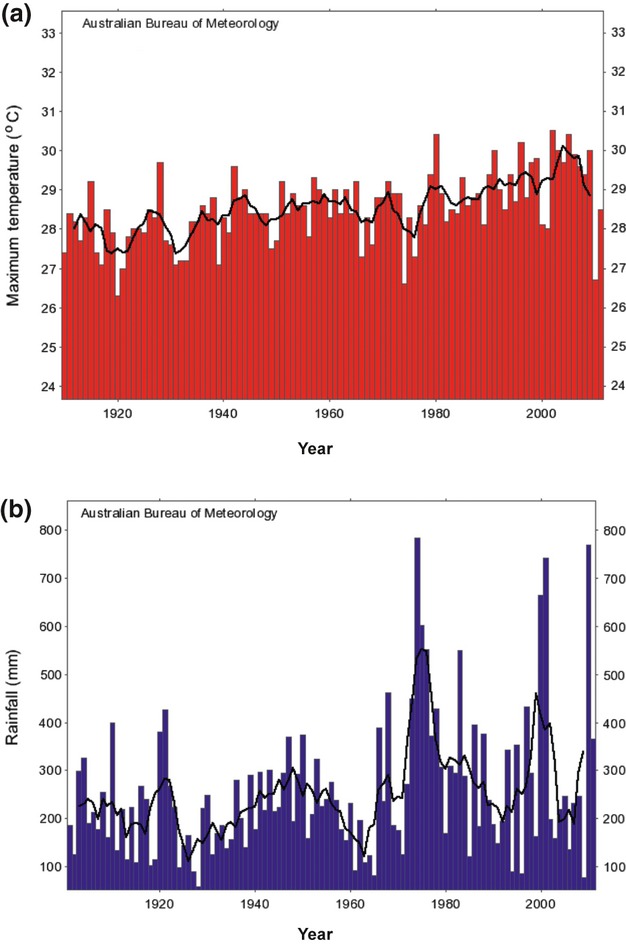
Time series with a 5 year moving average (continuous line) of: (a) annual maximum temperature; and (b) annual rainfall, Alice Springs, Northern Territory (23.8°S, 133.89°E). Source: http://www.bom.gov.au.

Here we combine arid with semiarid regions, which together form a coherent biome (Byrne *et al*., 2008) (Fig. 1). The biome is characterized by low and unpredictable annual precipitation (100–500 mm yr^−1^) and extremely high potential evaporation (2880–4000 mm yr^−1^) (http://www.bom.gov.au). It is the largest biome on the continent, occupying approximately 70% of the landmass of 7.5 million square kilometres, and represents one of the largest desert landforms in the world (Byrne *et al*., 2008). It is a region of low relief (<300 m above sea level) with the exception of the Pilbara–Hamersley Ranges in Western Australia, the Central Ranges in the Northern Territory and the Flinders Ranges in South Australia. Some of the most rapid climate warming recorded since European colonization of the Australian continent has occurred within the arid zone (Brim Box *et al*., 2008). Annual maximum temperatures recorded at the Alice springs meteorological station have increased by 2 °C since 1900 while annual rainfall remains highly variable, unpredictable and episodic (Fig. 2) (http://www.bom.gov.au).

## History of aridification

The climatic history of the Australian continent has been influenced by its movement northward (from 76°S to warmer latitudes) since the breakup of Gondwana during the Cretaceous (95 mya). The continent moved from 70 to 40°S during the Tertiary and continues to drift (at 20 mm yr^−1^) with its current position spanning 43–10°S [Veevers (1984) cited in Unmack (2001a]. Much of the continent's landmass now occurs at the earth's mid latitudes (approximately 30°S) where aridity dominates due to the sinking of hot, dry air associated with the subtropical, high pressure atmospheric belt.

Seasonal aridity developed first in northwestern Australia in the late Palaeocene (55 mya) moving further across the continent throughout the Miocene (Martin, 1998). Byrne *et al*. (2008) suggested that aridification began in the mid-Miocene (approximately 15 mya) but that the desert landforms appeared much later, approximately 1–4 mya. Dated molecular phylogenies of diverse arid-adapted taxa showing their deepest divergences from the mid-Miocene, indicate the likely onset of aridification (Byrne *et al*., 2008). Warmer and wetter conditions were present in the Pliocene (6–2.5 mya) followed by major oscillations between glacial and interglacial climates in the Pleistocene [2.5 mya–400 000 years (Byrne *et al*., 2008)].

The major glaciations recorded in the Northern Hemisphere during the Pleistocene did not occur in Australia: instead the continent became increasingly drier with extreme aridity occurring during global glacial cycles. Arid environments expanded during glacial intervals, although the interglacials may also have been relatively dry. Climatic oscillations peaked over the last 400 000 years, with widespread and extreme aridity occurring during the LGM. The Australian biota has experienced filters of increasing aridity throughout the Quaternary, with precipitation in the present interglacial phase not exceeding previous phases (Morton *et al*., 2011).

## Hydrological changes associated with aridification

Australia's long history of geological stability and lack of extensive ice-cover during Pleistocene glacial episodes had a major influence on the continent's aquatic ecosystems (De Deckker, 1986). The last major uplift, the formation of the Great Dividing Range (Fig. 1) along the eastern coastline, occurred 90 mya, and all major contemporary river basins were established by the end of the Paleocene (Unmack, 2001a). The paleodrainage valleys in inland Australia contained active rivers at least until the Eocene (56–34 mya). Calcretes (limestone outcrops) formed within the paleochannels during the dry conditions of the Late Eocene to Early Oligocene (37–30 mya) (Leys *et al*., 2003). Active rivers as well as numerous freshwater lakes existed in paleovalleys of the Yilgarn craton of central Western Australia and other inland areas. The process of karstification (dissolution of limestone formations by water) occurring during this period may have provided the first subterranean habitats (as caves or groundwater) (Morgan, 1993). With the onset of aridity, from the Middle to Late Miocene (10 mya onward), the large inland freshwater lakes disappeared, the permanent rivers ceased flowing and salt lakes developed (Van Der Graaff *et al*., 1977). Calcrete aquifers were sustained by regional aquifers and locally by recharge from episodic rainfall (Morgan, 1993). These aquifers probably became available as subterranean aquatic habitats long before aquatic conditions above ground became unfavorable (Leys *et al*., 2003). The springs fed by the Great Artesian Basin (GAB) originated approximately 1 mya following the formation of deserts approximately 2–4 mya (Prescott & Habermehl, 2008).

The past 50 000 years of the hydrological history of lakes and rivers in southeastern Australia is relatively well understood, based on records of lacustrine sedimentation (De Deckker, 1986). Hydrological conditions fluctuated during this period, with the most extreme aridity and drying of lakes corresponding with the LGM. The aquatic biota that survived the LGM either dispersed to coastal waterbodies (that were later inundated by rising seas) or remained within refugia containing permanent water (De Deckker, 1986).

## Classification of arid zone aquatic habitats

**Fig. 3:**
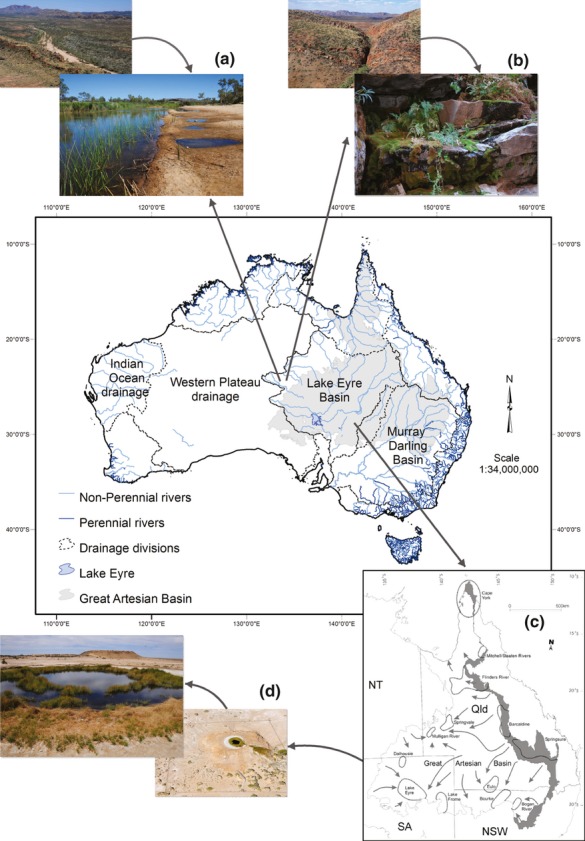
Location of Australia's major river systems and the major arid zone drainage basins: the Lake Eyre Basin, the Murray-Darling Basin (part); the Western Plateau and the Indian Ocean drainage divisions. Source: http://www.bom.gov.au: (a) aerial and ground view of an ecological refuge, Two Mile Waterhole, the Finke River, West MacDonnell Ranges, Northern Territory (23.40°S,132.40°E); (b) aerial and ground view of an evolutionary refugium, Serpentine Gorge, West MacDonnell Ranges, Northern Territory (23.45°S,132.36°E); (c) the Great Artesian Basin showing recharge zones (shaded), spring supergroups (dotted lines), and flow direction (arrows). Source: http://wetlandinfo.derm.qld.gov.au; (d) aerial and ground view of an evolutionary refugium, The Bubbler, a discharge spring in the Lake Eyre South group, South Australia (29.45°S, 136.87°E).

The central role of habitat in the definition of refugia (Keppel *et al*., 2012) suggests that classifying arid zone aquatic habitats is a useful first step for identifying aquatic refugia. The Australian arid zone contains freshwater ecosystems of varying depths, areas and water quality, surrounded by vast tracts of arid land. Extensive river networks and springs are largely confined to the Lake Eyre Basin (LEB) and the Pilbara-Hamersley region of Western Australia (Fig. 3). Aquatic habitats sustained by groundwater are also present within subterranean aquifers (Humphreys, 2006). Four types of permanent arid zone waterbodies: riverine waterholes, rockholes, discharge springs and outcrop springs, were described for the eastern LEB by Fensham *et al*. (2011). Riverine waterholes were the most common and widespread, while springs and rockholes were confined to relatively discrete clusters. The classification of Fensham *et al*. (2011) recognized the major geomorphic attributes of these systems, which, being fixed or structural attributes of geology and landform, are much less variable than water quantity or quality. We extended the Fensham *et al*. (2011) classification across the entire arid zone biome to include subterranean aquifers, relict streams, stream pools, isolated rockholes, claypans and temporary lakes, and summarized the main characteristics of each as follows.

### Subterranean aquifers

Groundwater calcrete aquifers are the most widespread of subterranean aquatic habitats occurring mainly through the central region of Western Australia. The calcretes are discrete, shallow and thin (10–20 m thick) carbonates deposited from the groundwater flow in paleovalleys immediately upstream of salt lakes. Voids in fractured rock aquifers in the Pilbara-Hamersley region also support aquatic organisms (stygofauna) (Humphreys, 2006). Arid land subterranean aquatic habitats have greater hydrological stability than do surface water habitats. They are characterized by a lack of light, and associated lack of primary production, and often exhibit marked gradients in organic carbon, oxygen, salinity, Eh, pH and water chemistry along groundwater flowpaths and at depth within the aquifer (Humphreys, 2006).

### Discharge springs

These are surface expressions of a major regional aquifer, the Great Artesian Basin (GAB), which covers 22% of the Australian continent and is one of the world's largest artesian systems (Prescott & Habermehl, 2008) (Fig. 3). The springs arise through fault structures, where the aquifer adjoins protrusions of basement rock, or where the confining beds are sufficiently thin to allow discharge. Often the springs, also known as mound springs, sustain small permanent downstream wetlands (Fensham *et al*., 2011). Discharge springs arise in areas remote from where the aquifer receives its input, mainly on the GAB margins (Fig. 3). Long groundwater residence times result in consistent flows and alkaline waters with high concentrations of dissolved solids (Fensham *et al*., 2011).

Both discharge and outcrop springs (described below) are clustered at a range of scales, from individual vents to spring complexes to ‘super-groups’ at regional scales. The ‘spring complex’ scale is defined as a group of springs where no adjacent pair of springs is more than 6 km apart and all springs within the complex are in a similar geomorphic setting (Fensham *et al*., 2011). Thirteen major supergroups of spatially clustered spring complexes are recognized (Ponder, 2002) (Fig. 3). They represent naturally highly fragmented ecosystems where the springs form aquatic ‘islands’ of biodiversity in a ‘sea’ of inhospitable desert. Floods can result in occasional hydrological connectivity between springs that are located within the same drainage basin.

### Outcrop springs

These are habitats that are entirely dependent on groundwater from local aquifers and occur where sediments forming the aquifer outcrop at the surface. The groundwater in these local aquifers can have relatively short residence times and some springs contract to seepages or disappear completely in very dry times (Brim Box *et al*., 2008). The water is slightly acidic (comparable to rainwater) with low concentrations of dissolved solids (Fensham *et al*., 2011). The majority of springs in central Australia discharge from fractured rock aquifers of sandstone, fissured limestone or quartzite, and are usually located near the base of ranges.

### Relict streams

These are small, permanently flowing sections of streams in the headwaters of the now mainly dry river systems of the Central Ranges. Local flows occur due to the presence of springs arising from local fractured rock aquifers. They are relatively cool, mesic-type habitats: most occur within deeply shaded, south-facing gorges (Fig. 3). The spring waters are very fresh (often <100 μS cm^−1^) with stable ionic concentrations because shading lessens the evapoconcentrative effects that predominate in more exposed rockholes and riverine waterholes. The term ‘relict stream’ was first applied by Davis *et al*. (1993) in recognition of the populations of stream-dwelling insects with Gondwanan affinities that occur in these habitats, but nowhere else in the rarely flowing river networks of the Central Ranges. Although these sites can be initially recognized on the basis of geomorphic features they can only be confirmed by presence of permanent groundwater and taxa with a requirement for shading and cooler thermal regimes. Surveys based on the presence of specific ferns (P. Latz, Personal communication) and aquatic insects (Coleoptera: Psephenidae) have identified eight relict stream sites within the Central Ranges (Davis, 1997).

### Riverine waterholes

These are the largest and deepest waterbodies that occur throughout the once extensive but now dry river networks of the Australian arid zone. They are connected when large, infrequent rain events result in high flows or flooding (Fig. 3). Between flows they become disconnected and contract in area and depth. Surface water inputs and evaporative processes dominate, although some are sustained by local groundwater inflows (Hatton & Evans, 1998). Riverine waterholes can range from deep, permanent pools to shallow and temporary or very ephemeral pools. They range from 50 m to 20 km in length, with depths >4 m needed to maintain permanent water (Costelloe *et al*., 2007; Fensham *et al*., 2011). Riverine waterholes in the eastern LEB are often turbid (Bunn *et al*., 2006) while those in the western LEB and Pilbara region are often clear, although smaller creeks can contain highly turbid pools (Pinder *et al*., 2010).

### Stream pools

Episodic rainfall events flood small, rocky headwater creeks in arid zone ranges to create pools (also known as rockholes). Although most are temporary, some exist as permanent or near-permanent pools. They differ from relict streams and outcrop springs in that they are dominated by surface water. They differ geomorphically from riverine waterholes; stream pools occur within first and second order river networks while riverine waterholes are located in higher order network sections. Rainfall-induced flow events support short-lived hydrological connectivity along streamlines.

### Isolated rockholes

These are natural hollows, formed by fracturing and weathering of rocky landscapes, which store water from infrequent local run off (Bayly, 2001; Fensham *et al*., 2011). These are surface water-dominated rather than groundwater-dependent systems. They are the smallest and most isolated of arid zone waterbodies, and are widespread, although not abundant, throughout the region. The pools on granite outcrops in Western Australia represent a subset of this category.

### Claypans

These are small, temporary, shallow basins fed by local run off and dominated by evaporative processes. They contain fresh and characteristically turbid water.

### Lakes

These are large, shallow, isolated basins with highly variable (temporary to ephemeral) and unpredictable hydroperiods. They are fed by local runoff after infrequent rain events. Extensively vegetated lake systems are often called swamps or marshes.

## Identification of evolutionary refugia and ecological refuges

We assessed the likely role of arid zone aquatic habitats as evolutionary refugia (based on millennial timescales) by firstly considering the phylogeny (evolutionary history), particularly the time of divergence, of dominant faunal groups (Table 1). Highly divergent lineages are considered to provide evidence of long-term isolation and persistence (Byrne *et al*., 2008). Refugia can also be identified by the presence of relict species (taxa possessing ancestral characteristics) and short-range endemics (SRE's) *sensu* Harvey (2002), taxa with highly restricted distributions that occupy a very small area. These include taxa that were formerly more widespread but now occupy much smaller areas and others that are restricted to specific habitats. Important life history traits of SRE's include poor powers of dispersal, confinement to discontinuous or rare habitats, slow growth and low fecundity (Harvey *et al*., 2011).

**Table 1:** Attributes supporting the determination of arid zone evolutionary refugia. NR = Not Recorded

Aquatic habitat	Divergence time of dominant fauna	Short-range endemics present	Relictual species present	Sources	Likely importance as evolutionary refugia
Subterranean aquifers	Mid-Miocene 3–11 mya	Yes	Yes	1–5	High
Discharge springs (GAB)	2.5–0.4 mya	Yes	Yes	6–8	High
Outcrop springs	Unknown	NR	NR		Low
Relict streams	LGM	Yes	Yes	9	High
Riverine waterholes	2.5 mya-present approximately varying times for different taxa (Mollusca, Crustacea and fishes)	Regional endemism detected in some fish taxa	No	110–16	Moderate for permanent & low for temporary waterholes
Stream pools	Unknown	NR	NR	9	Low
Isolated rockholes	Unknown	NR	NR	17	Low
Claypans	Unknown	NR	NR		Low
Temporary lakes	Unknown	NR	NR		Low

Sources: (i) Byrne *et al*. (2008); (ii) Leys *et al*. (2003); (iii) Cooper *et al*. (2007, 2008); (iv) Humphreys (2006); (v) Guzik *et al*. (2010); (vi) Perez *et al*., 2005; (vii) Murphy *et al*. (2009, 2012); (viii) Worthington Wilmer *et al*. (2008); (xi) Davis *et al*. (1993); (x) Murphy & Austin, 2004; (xi) Carini & Hughes (2004, 2006); (xii) Hughes *et al*. (2004); (xiii) Hughes & Hillyer (2003, 2006); (xiv) Nguyen *et al*. (2004); (xv) Bostock *et al*. (2006); (xvi) Unmack (2001a, b); and (xvii) Bayly (2001).

Subterranean aquifers contain communities of obligate groundwater taxa that represent genetic diversity isolated underground during different geological periods (Humphreys, 2006; Guzik *et al*., 2010). Crustacean lineages persisting since the breakup of Gondwana include the phreatoicidean and tainisopidean isopods, crangonyctoid amphipods and candonine ostracods. The bathynellaceans are considered to have persisted since Pangaea. Dytiscid diving beetles and oniscidean isopods are believed to have invaded inland groundwaters during the Tertiary (Humphreys, 2006).

Population genetic analyses of the subterranean dytiscids, *Paroster macrosturtensis, P. mesosturtensis* and *P. microsturtensis* provide evidence for multiple expansion events within each species (Guzik *et al*., 2009). A subsequent comparative study of the genetic diversity and population genetic structure of these dytiscid beetle species, one chiltoniid amphipod species and a lineage of *Haloniscus* isopods by Guzik *et al*. (2011) revealed a shared evolutionary history among these subteranean species. They considered it likely that multiple isolation and expansion events had occurred at different times within the study aquifer. The presence of phyletic relictual species, as well as higher taxa typically comprising short-range endemic species, and evidence of population expansion and contraction, suggest that the subterranean aquatic habitat can be classified as an evolutionary refugium.

The evolutionary importance of discharge springs (mound springs) is well documented. The springs support endemic and relict species, dominated by hydrobiid molluscs and crustaceans (including isopods, amphipods and ostracods), with limited mobility and dispersal potential (Ponder, 2003; Perez *et al*., 2005; Worthington Wilmer *et al*., 2008; Murphy *et al*., 2009, 2012). A molecular study of hydrobiid snails found that at least three separate colonization events of GAB discharge springs had occurred between 2.5 and 0.4 mya (Perez *et al*., 2005). Determination of the time-scale of population divergence of the hydrobiid genus *Trochidrobia* found similar results, suggesting that increased periods of aridity and the formation of inland deserts had led to multiple *Trochidrobia* species becoming ‘trapped’ in desert spring refugia with no subsequent gene flow between populations (Murphy *et al*., 2012). A phylogeographic study of chiltoniid amphipods found evidence of multiple independent colonizations, particularly within the Lake Eyre group of springs (Murphy *et al*., 2009). Evidence of a shared evolutionary history between these and Western Australian subterranean amphipods (up to 1500 km away) and approximate dating of the diversity found between major clades suggested that the majority of lineages originated in the late Miocene, coincident with the onset of aridification. Fish species recorded from the springs represent both endemics of uncertain origin with presumed limited dispersal capacity (Harris, 1992) and widespread species of presumed higher mobility (Wager & Unmack, 2000).

Davis *et al*. (1993) recognized the evolutionary importance of groundwater-fed, shaded headwater stream habitats in the Central Ranges based on the disjunct distributions of stream-dwelling aquatic insects, including the water penny *Sclerocyphon fuscus*, the mayfly *Atalophebia australis* and the caddisflies, *Hellyethira simplex* and *Ecnomus continentalis*. The low vagility of the adults of these species suggested that they would not be capable of dispersal across the thousands of kilometres of arid land that separate populations in central Australia from the streams where they also occur in southeastern Australia. They suggested that these taxa may have dispersed during a pluvial phase of the Quaternary before the LGM, rather than earlier in the Tertiary, based on a reconstruction of the phylogeny of the genus *Sclerocyphon* by Davis (1986).

Permanent riverine waterholes act as long-term refuges for fish and fully aquatic invertebrates during dry periods, with long-term population persistence dependent on dispersal and recolonization among waterholes during periods of high flow (Arthington *et al*., 2005). Three major types of dispersal strategies have been identified in fragmented dryland river landscapes: ‘movers’, the organisms that have mobile adults and do not require physical flow connectivity to disperse across waterholes (e.g. Odonata, Heteroptera, Coleoptera); ‘networkers’, the organisms that disperse easily during the high flow through the channel network (e.g. Crustacea, some fish); and ‘permanent refugial’ organisms (primarily Mollusca) that have limited dispersal abilities even under flow conditions (Sheldon *et al*., 2010). The role of riverine waterholes as ecological refuges is well established, but their role as evolutionary refugia needs further investigation.

Molecular studies have detected significant levels of genetic diversity, both among waterholes within the Lake Eyre and Murray–Darling drainages and between drainages for four species of freshwater mussels, *Velesunio* spp. (Bivalvia : Hyriidae) (Hughes *et al*., 2004), a freshwater snail, *Notopala sublineata* (Gastropoda : Viviparidae) (Carini & Hughes, 2006), a crayfish, *Cherax destructor* (Decapoda : Parasticidae) (Hughes & Hillyer, 2003) and a freshwater prawn, *Macrobrachium australiense* (Decapoda : Palaemonidae) (Carini & Hughes, 2004). The large scale but infrequent (approximately 10 year) flooding of the ‘boom and bust’ cycles in the Lake Eyre system (Puckridge *et al*., 1998) resulted in less mixing of individuals than would be expected. This is because strong swimmers may actively enter flood currents while weaker swimmers may seek to avoid flood currents and so tend to remain in their waterhole of origin (Hughes & Hillyer, 2006). The importance of these waterholes as evolutionary refugia is likely to be species-specific, indicating the need for more extensive genetic studies to fully determine the extent of isolation of populations of dominant waterhole taxa.

Fishes, which comprise a major component of the biomass of riverine waterholes, are stronger swimmers than most aquatic invertebrates and are known to move out from waterholes onto floodplains during floods (Puckridge *et al*., 1998). Accordingly, they would be expected to display higher levels of connectivity/gene flow than obligate aquatic invertebrates, but not those with a flying life history phase. Allozyme and mitochondrial DNA data for two species of freshwater fishes, the bony bream *Nematolosa erebi* (Clupeidae) and the Australian smelt *Retropinna semoni* (Retropinnidae) revealed that while there was no contemporary dispersal across the Lake Eyre and Murray–Darling drainage boundaries, there was evidence of historical connections. However, *N. erebi* populations from the two drainages were estimated to have separated ca. 150 000 years ago, whereas populations of *R. semoni* were estimated to have separated ca. 1.5 Ma (Hughes & Hillyer, 2006) suggesting different timescales exist for different species with respect to the waterholes acting as refugial habitats. Further studies of the population genetics of arid zone fishes with different dispersal traits (fast vs. slow dispersers) are needed to more fully determine the role of riverine waterholes as evolutionary refugia and ecological refuges.

Unmack (2001a) investigated the biogeography of the Australian freshwater fish fauna by looking for congruent distributional patterns among species. Connectivity was inferred where species were shared between drainages. Biogeographic patterns were hypothesized and compared with geological and climatic records. Low species richness within some arid regions was attributed to lack of water, and high richness (30 spp) in the LEB to its large area and the presence of discharge springs. The high regional endemism found in the Pilbara (42%) and the LEB (40%) were considered to represent divergence arising in the Miocene-Pliocene. In contrast, Bostock *et al*. (2006) found very limited genetic diversity within one of the most widespread species, the spangled perch, *Leiopotherapon unicolor,* (Terapontidae) suggesting that it is a very effective disperser, had gone through a genetic bottleneck and had probably achieved its current distribution from the Pleistocene to the present.

**Fig. 4:**
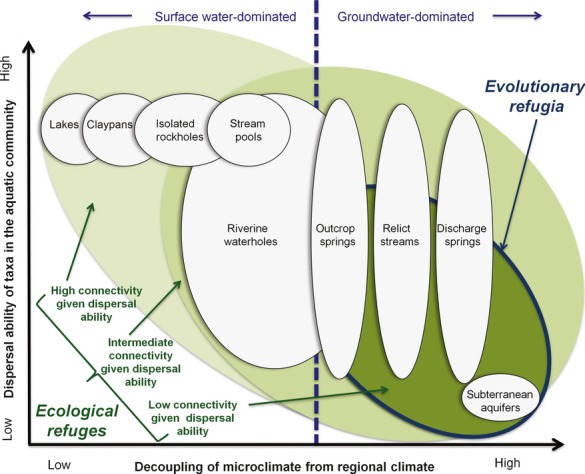
Conceptualization of the major differences between evolutionary refugia (aquatic habitats supporting populations with low dispersal ability but high climatic decoupling) and a range of ecological refuges (aquatic habitats supporting populations with varying dispersal abilities and exposure to ambient climatic processes). Aquatic habitats with the greatest degree of decoupling of microclimate from regional climate are the most likely to act as evolutionary refugia. Those with the least decoupling function as ecological refuges for only the most mobile of aquatic taxa. Some habitats potentially act as both an evolutionary refugium and an ecological refuge, depending on the dispersal traits of the taxa they support, their geographical proximity and hydrological connectivity.

For obligate aquatic organisms (all fish and some aquatic invertebrates, e.g. large crustaceans and molluscs) perennial aquatic systems are the most likely to function as ecological refuges (Fig. 4). They are the habitats where aquatic organisms can persist during extended periods without rain. In contrast, the temporary and ephemeral waterbodies that arise as a response to rain events but are short-lived (containing water for only days, weeks or months) are likely to act as refuges for only very mobile taxa (the robust flying adult stages of aquatic insects such as dragonflies and beetles) (Fig. 4). Some perennial sites can act as both a refugium for species with low dispersal capabilities and a refuge for more mobile species. Perennial waterbodies support meta-population dynamics by providing ‘reservoirs’ from which individuals can disperse. Temporary aquatic habitats play important roles as ‘stepping stones’ between more permanent sites and by providing extra resources that enable populations to increase, reproduce and replenish egg and seed banks during wet phases (booms) (Sheldon *et al*., 2010). Hydrological connectivity is putatively maintained in aquatic habitats located within dryland river networks, but only at the very low occurrence frequencies of large episodic rain events. In contrast, hydrological connectivity is absent (or restricted to very small spatial scales) in isolated standing water and subterranean habitats (Table 2). Accordingly, we hypothesize that the extent of gene flow in species that can disperse only within water (all fishes and some aquatic invertebrates) will be restricted to very small spatial scales in isolated standing-water habitats, but extend over much larger, watershed scales in river networks. Gene flow in populations of strong aerial dispersers, such as the Odonata (Aeshnidae, Corduliidae, Gomphidae, Hemicorduliidae and Libellulidae), Coleoptera (Dysticidae, Gyrinidae and Hydrophilidae) and Heteroptera (Notonectidae and Corixidae), will potentially occur at the much larger spatial scales of the entire arid biome or the continent. Gene flow in weaker aerial dispersers, such as Ephemeroptera (Baetidae, Caenidae and Leptophlebiidae) and Trichoptera (Ecnomidae, Leptoceridae and Hydropsychidae), is likely to occur at intermediate (regional) or local scales.

**Table 2:** Putative hydrological connectivity and scales of gene flow associated with the fauna (fishes and aquatic invertebrates) of arid zone aquatic habitats

Aquatic habitat	Type of refugium/refuge	Putative hydrological connectivity	Putative scale of gene flow *Aquatic Dispersers*	Putative scale of gene flow *Aerial Dispersers*
Subterranean aquifers	Evolutionary	Low	Local	NA
Discharge springs (Great Artesian Basin)	Evolutionary & ecological	Low	Local	Biome
Outcrop springs	Ecological & ecological	Low	Local	Biome
Relict streams	Evolutionary & ecological	Moderate	Local	Regional
Riverine waterholes	Evolutionary & ecological	High	Watershed/drainage basin	Drainage basin/biome
Stream pools	Taxa and context dependent	High	Watershed	Biome
Isolated rockholes	Taxa and context dependent	None	None	Biome/continent
Claypans	Taxa and context dependent	None	None	Biome/continent
Temporary lakes	Taxa and context dependent	None	None	Biome/continent

## The influence of climate and hydrology on the persistence of arid zone aquatic refugia and refuges

To determine the likely persistence of refugia and refuges in the face of anthropogenic climate change, we examined the environmental processes supporting the formation and maintenance of aquatic habitats with the specific aim of identifying where local processes are decoupled from regional processes, as advocated by Dobrowski (2011) (Table 3). All habitats supported by groundwater are at least partially decoupled from local rainfall. Subterranean aquatic habitats are the most highly decoupled from arid zone precipitation and temperature regimes. Most are supported by groundwater that has accumulated over much longer timescales than that of annual rainfall, and their underground location acts as a buffer from the extreme highs and lows of desert temperatures.

**Table 3:** Major hydrological and climatic attributes of Australian arid zone aquatic habitats and the likely extent of climatic decoupling. GW = groundwater, SW = surface water, GAB = Great Artesian Basin. P = perennial hydroperiod (permanent aquatic habitat), I = intermittent hydroperiod (temporary or ephemeral aquatic habitat)

Aquatic habitat	Dominant water source	Hydroperiod	Hydrological variability	Sources	Likely decoupling from regional climate (Rainfall)	Likely decoupling from regional climate (Temp)
Subterranean aquifers	GW	P	Low	1	High	High
Discharge springs (GAB)	GW	P	Low	2, 3	High	Low
Outcrop springs	GW	P/I	Low	2, 3	Moderate	Low
Relict streams	GW	P	Moderate	3, 4	High	Moderate
Riverine waterholes	SW/GW	P/I	High	2, 3, 4, 5, 6	Moderate/Low	Low
Stream pools	SW	I	High	4, 6	Low	Low
Isolated rockholes	SW	I	High	7	Low	Low
Claypans	SW	I	High	8, 9	Low	Low
Temporary lakes	SW	I	High	8, 9	Low	Low

Sources: (i) Humphreys (2006); (ii) Fensham *et al*. (2011); (iii) Brim Box *et al*. (2008); (iv) Davis *et al*. (1993); (v) Bunn *et al*. (2006); (vi) Unmack (2001b); (vii) Bayly (2001); (viii) Roshier *et al*. (2001); and (ix) Boulton & Brock (1999).

The discharge springs, fed by the GAB (a very large aquifer that is mainly recharged from a much wetter climatic zone on the eastern margin of the continent) are the most highly decoupled, spatially and temporally, from annual rainfall (Fig. 3). However, because these springs lack shading by either topographic features or a well-developed canopy of fringing vegetation (as illustrated by the image of a typical representative of a discharge spring in Fig. 3), they are fully exposed to contemporary thermal regimes.

The relict streams present within the Central Ranges, fed by local aquifers, are decoupled from annual rainfall but on shorter timescales (residence time approximately 100 years) than are the discharge springs supported by the GAB. Their location within shaded gorges also provides decoupling from ambient temperatures (Fig. 3). In contrast, outcrop springs (by definition also supported by local aquifers) are similarly decoupled from annual rainfall, but are not topographically shaded, and so are exposed to ambient temperatures.

Riverine waterholes and stream pools/rockholes are fully exposed to the arid regional climate. Sparse riparian vegetation provides little shelter from the extremes of inland continental temperature regimes (Fig. 3). Large episodic rainfall events are the dominant source of water, resulting in extremely high hydrological variability. Permanent, deep riverine waterholes may also be partially sustained by groundwater held in local aquifers, providing some degree of hydrological buffering under low flow conditions and some thermal buffering, particularly where thermal stratification occurs.

Isolated rockholes, claypans and lakes are usually fully exposed to the regional climate, as they are dependent upon surface water runoff generated by infrequent local precipitation and, unless very deep, are poorly buffered from local temperatures.

## Likely future refugia and vulnerability under climate change

The ability of refugia to mitigate locally the effects of regional climate change is increasingly considered important for climate change adaptation planning (Ashcroft, 2010; Keppel & Wardell-Johnson, 2012). Including refugia in protected area networks and in climate change management planning should be a high priority because they offer the only hope for *in situ* persistence of poorly dispersed species (Game *et al*., 2011).

Perennial aquatic habitats (subterranean aquifers, discharge springs, relict streams and permanent riverine waterholes) that currently act as evolutionary refugia for vicariant relicts and SRE's are likely to persist as future refugia because local population sizes are large enough to maintain viable populations over very long periods. However, these populations are also extremely vulnerable because a change in local conditions leading to population decline or extinction cannot be remedied by dispersal of individuals from other sites. These habitats will persist as evolutionary refugia if local climate decoupling is sufficient for biota to experience acceptable environmental conditions. They will also continue to act as ecological refuges for more mobile taxa. Habitats with more variable hydroperiods (outcrop springs, temporary riverine waterholes, stream pools, isolated rockholes, claypans and temporary lakes) contain biota supported by contemporary demographic and genetic (demogenetic, *sensu* Frank *et al*. (2011)) processes. These species will persist where suitable habitats are available and dispersal pathways are maintained. These systems are governed by the expansion and contraction dynamics described by Stanley *et al*. (1997) and the boom and bust dynamics described by Kingsford *et al*. (1999), Bunn *et al*. (2006) and others. Gene flow will occur over a range of scales, depending on the dispersal traits and population sizes of individual species coupled with hydrological connectivity (Bohonak & Jenkins, 2003). Maintaining connectivity by mitigating barriers to dispersal and alterations to the natural flow regime are recommended as important management strategies for arid zone fishes (Faulks *et al*., 2010). These strategies will also help conserve arid zone riverine invertebrates.

Groundwater-dominated habitats have a theoretically higher likelihood of persistence under future climate change than surface water-dominated habitats because they are largely independent of local precipitation. However, aridification over millennia means that groundwater systems in arid Australia are in a state of net discharge, not equilibrium (Hatton, 2001). Accordingly, the habitats and species supported by groundwater are highly vulnerable to anthropogenic impacts, especially drawdown of groundwater for agriculture and mining.

Discharge (mound) springs, which are decoupled from local precipitation but not local temperatures, and support species for which demogenetic processes are restricted to local scales (from m to km) (Worthington Wilmer *et al*., 2008) appear to be the most vulnerable to climate warming. Although subterranean aquifers and relict streams appear to be more persistent habitats because they are decoupled from low arid zone precipitation and extreme thermal regimes, their dominant species are highly vulnerable to extinction because populations will not be replaced by dispersal from other sites. All temporary and ephemeral aquatic habitats have lower hydrological persistence and are fully exposed to contemporary arid zone climatic processes. However, the biota that inhabit them appear to be far less vulnerable to extinction because they have dispersal mechanisms that facilitate meta-population dynamics and gene flow over much larger spatial scales. If climate change affects dispersal processes, the responses of individual species may vary, depending upon their dispersal abilities and gene flow.

Comparison of different types of aquatic ecosystems based on their decoupling from the regional climate (temperature and rainfall) with the varying dispersal abilities of arid zone taxa (Fig. 4) illustrates the major differences between evolutionary refugia (restricted gene flow but high climatic decoupling) and a range of ecological refuges (high gene flow but exposed to ambient climatic processes). Systems at one extreme (climatically decoupled, perennial hydroperiods) are either a refugium or refuge depending on the evolutionary history, gene flow and dispersal abilities of their taxa and their geographical proximity and hydrological connectivity. Systems at the other extreme (climatically coupled, temporary and highly variable hydroperiods) are rarely likely to act as major refuges for all but a small number of highly mobile taxa.

Further investigations of meta-population dynamics and gene flow in arid zone aquatic species, particularly aquatic invertebrates with different dispersal modes (active, weak and passive), are needed to more fully describe our habitat categories and the genetic processes associated with their dominant species. A number of species recorded from evolutionary refugia appear to correspond to the Death Valley Model of extreme isolation and high-within and low-between population gene flow (Meffe & Vrijenhoek, 1988). Some species associated with ecological refuges are more likely to accord with the Stream Hierarchy Model where population structure is more complex and is influenced by geographical proximity and connectivity of habitats. Meffe & Vrijenhoek (1988) noted that the two systems must be recognized as being distinct and requiring different types of management.

Some factors that may influence habitat characteristics and associated biotic patterns may not be recognized by placing habitats into discrete categories (Fig. 4). The advantage of defining habitats as evolutionary refugia or ecological refuges is that it provides guidance to managers as to how these sites may be conserved based on the most influential commonalities and distinctions.

We have only qualitatively identified refugia and refuges as generalized habitat types and further work is needed to produce a quantitative assessment of the spatial distribution of these types. This could be undertaken using methods similar to those of Fensham *et al*. (2011) that included analysis of satellite imagery and interviews with long-term land managers. The large variation in some habitat characteristics, that is, the relative contributions of groundwater and surface water, and the amount of topographic shading, needs to be determined for some habitats (particularly riverine waterholes and outcrop springs) to inform potentially different management approaches within habitat types. The large spatial extent of the Australian arid biome suggests that some environmental factors will vary significantly across the biome. For example, although aridity is the major environmental driver, other factors such as the timing of major rainfall events, from predominantly summer in the north to winter in the south, may influence the capacity of some waterbodies to act as refugial habitats.

## Approaches to climate change adaptation and conservation

Climate change adaptation is defined as the adjustment of natural or anthropogenic systems to a changing climate for the purpose of moderating impacts or capitalizing on novel opportunities (IPCC, 2007b). Groves *et al*. (2012) proposed five approaches to climate change adaptation that can be integrated into existing or new biodiversity conservation plans. These included the following: (i) conserving the geophysical stage; (ii) protecting climatic refugia; (iii) enhancing regional connectivity; (iv) sustaining ecosystem process and function; and (v) capitalizing on opportunities emerging in response to climate change. A major strength of these approaches is that they are generally robust to the uncertainty in how climate impacts may manifest in any given location.

In this article we have distinguished between aquatic habitats that are likely to act as climatic refugia (evolutionary refugia) because of decoupling from the regional climate (rainfall) and other habitats that will not, but have importance with respect to enhancing regional connectivity and sustaining ecosystem processes and functions.

Mawdsley *et al*. (2009) proposed 16 adaptation strategies grouped into four broad categories: land and water protection and management; direct species management; monitoring and planning; and law and policy. Although many of these can be considered ‘business as usual’, these strategies will increasingly need to be viewed through the ‘lens of climate-induced changes to species and ecosystems’. For example, the inclusion of evolutionary refugia and major ecological refuges within regional reserve networks represents an important conservation strategy, but it will have additional adaptation benefits if the groundwater and surface water resources that support them are also identified and protected. The fundamental importance of groundwater in maintaining evolutionary refugia suggests that mapping and protection of their aquifers is an adaptation action of the highest priority. Important attributes for prioritizing the protection of ecological refuges include knowledge of their geographical proximity and degree of connectivity. In the absence of hydrological data, and for spatially isolated waterbodies, this will be provided by obtaining information on the genetic structure of populations of representative species of fish and aquatic invertebrates.

Implementing conservation programs that are good for biodiversity, regardless of future climates, represent valuable ‘no-regrets’ actions (Groves *et al*., 2012). For arid zone aquatic systems worldwide, this includes reducing existing stressors such as the overextraction of aquifers, the impacts of invasive species and habitat degradation. In some cases, these impacts may far exceed those of climatic change. Emerging opportunities also need to be considered as part of a broad, strategic approach to climate adaptation planning (Groves *et al*., 2012). New and novel waterbodies created by arid zone industries (e.g. mining) may represent valuable offsets for ecological refuges lost through climatic drying. These opportunities need to be recognized but the possible trade-offs for biodiversity need to be clearly articulated and rigorously assessed. This is especially important if the creation of new waterbodies has occurred by extracting water from aquifers that support evolutionary refugia.

The establishment of a biome-wide monitoring program, incorporating relatively inexpensive and portable weather stations, aquatic loggers and sampling of key taxa at representative refugial sites, is recommended as part of a strategic adaptive management framework. The latter follows a generic process adopted by the international union for conservation of nature for protected area management based on: setting the ‘desired future condition’ and management options; operationalization; and evaluation and learning. This approach accounts for the complexity arising from interacting drivers that change over time (temperature, rainfall, flow, human skills capacity and levels of trust) and the interdependent behaviors of socio-ecological interactions (Kingsford *et al*., 2011). It appears well suited to the management of remote arid zone aquatic ecosystems that are located outside protected areas. The data collected will provide feedback on adaptation actions and information on the extent of decoupling of refugial microclimates from larger scale regional climatic change.

## Conclusions

Climate change adaptation requires strategies to increase resilience and resistance and to reduce vulnerability, exposure and uncertainty. This applies to species and their habitats, and the processes that support them. Our identification of different types of arid zone aquatic habitats as evolutionary refugia or ecological refuges are key concepts that can be used to guide arid zone climate adaptation policy and planning because they provide information on the vulnerability of these habitats to a changing climate, particularly a change in water regime, and the processes sustaining community persistence.

Strategies to increase resistance represent a major adaptation goal for arid zone evolutionary refugia where resistance implies the ability to withstand change, despite changing water availability. Evolutionary refugia contain relict and endemic species, which have a limited ability to persist in the absence of water, but can display high resistance to changing local conditions. These species are highly vulnerable to local extinctions and must be managed on a site-specific basis. Conservation planning requires a high level of protection for the aquifers that support refugial sites and water quality and habitat conditions must be maintained or restored at individual sites and ‘spring-groups’.

Strategies to increase resilience are essential for the conservation of ecological refuges. Here, resilience implies that a system or organism will change when water is scarce or disappears, but it will return to the previous state, when water returns. Accordingly, conservation of ecological refuges must focus on supporting dispersal, colonization and establishment processes, especially maintaining connectivity between waterholes in dryland river networks. This includes ensuring that surface flows are not impounded or diverted, or, if they are, that flows are managed to maintain the spatial and temporal connectivity essential to the persistence of priority aquatic taxa. Maintaining high quality habitats spanning the distributional ranges of priority taxa, and restoring degraded ecological refuges that are not well represented across the landscape, are also important climate adaptation actions.

Studies that seek to understand multiple, interacting processes (climatic, hydrological, ecological and demogenetic) are needed to further develop strategies to support the persistence of arid zone evolutionary refugia and ecological refuges in an era of unprecedented environmental change. Similar integrative studies which recognize the differences between evolutionary refugia and ecological refuges could also inform the conservation of aquatic and other habitats, and their associated biota, in other biomes.
